# The influence of fear of falling on the control of upright stance across the lifespan

**DOI:** 10.1016/j.gaitpost.2024.01.032

**Published:** 2024-01-29

**Authors:** M. Hill, L. Brayne, E. Hosseini, M. Duncan, T. Muehlbauer, S.R. Lord, T.J. Ellmers

**Affiliations:** a Centre for Physical Activity, Sport and Exercise Sciences, Coventry University, Coventry, United Kingdom; b Division of Movement and Training Sciences/Biomechanics of Sport, University of Duisburg-Essen, Essen, Germany; c Falls, Balance and Injury Research Centre, Neuroscience Research Australia, University of New South Wales, Sydney, New South Wales, Australia; d Department of Brain Sciences, Imperial College London, London, United Kingdom

**Keywords:** Postural control, Children, Age, Fear, Concern, Threat, Anxiety, Balance, Height

## Abstract

**Background:**

Standing at height, and subsequent changes in emotional state (e.g., fear of falling), lead to robust alterations in balance in adults. However, little is known about how height-induced postural threat affects balance performance in children. Children may lack the cognitive capability necessary to inhibit the processing of threat and fear-related stimuli, and as a result, may show more marked (and perhaps detrimental) changes in postural control compared to adults. This work explored the emotional and balance responses to standing at height in children, and compared responses to young and older adults.

**Methods:**

Children (age: 9.7 ± 0.8 years, n = 38), young adults (age: 21.8 ± 4.0 years, n = 45) and older adults (age: 73.3 ± 5.0 years, n = 15) stood in bipedal stance in two conditions: at ground level and 80 cm above ground. Centre of pressure (COP) amplitude (RMS), frequency (MPF) and complexity (sample entropy) were calculated to infer postural performance and strategy. Emotional responses were quantified by assessing balance confidence, fear of falling and perceived instability.

**Results:**

Young and older adults demonstrated a postural adaptation characterised by increased frequency and decreased amplitude of the COP, in conjunction with increased COP complexity (sample entropy). In contrast, children demonstrated opposite patterns of changes: they exhibited an increase in COP amplitude and decrease in both frequency and complexity when standing at height.

**Significance:**

Children and adults adopted different postural control strategies when standing at height. Whilst young and older adults exhibited a potentially protective “stiffening” response to a height-induced threat, children demonstrated a potentially maladaptive and ineffective postural adaptation strategy. These observations expand upon existing postural threat related research in adults, providing important new insight into understanding how children respond to standing in a hazardous situation.

## 1 Introduction

The influence of emotion (i.e., fear and anxiety) on human balance control systems is firmly established [1]. This has most frequently been studied through manipulations that increase the physical consequences of a loss of balance, by elevating the standing surface (typically to heights of between 0.6 to 3.2 m [1]). In these conditions, participants exhibit robust changes in balance, decreasing the amplitude and increasing the frequency of centre of pressure (COP) adjustments [1–12]. These changes have been interpreted as part of a postural “stiffening” response, whereby stiffness of the ankle joint is increased to tighten control of the centre of mass within the limits of the base-of-support [1,13,14]. Standing at height has also been shown to lead to greater ‘complexity’ (i.e., increased irregularity) in the COP signal in both young [15] and older adults [7].

Whilst postural control changes when standing at height are firmly established in both young (see [1] for a review) and older adults [7,8,10, 16,17], such postural responses have not yet been directly tested in children. Maturational and/or experiential immaturities in postural control mechanisms [18], and an emerging emotional processing capacity [19], may predispose children (6–12 years of age) to be more vulnerable to the effects of threatening stimuli on postural control. That is, when standing at height, children may lack the motoric and cognitive capability needed to inhibit the processing of threat-related stimuli, and, as a result, may show a pattern of potentially maladaptive behaviour more consistent with highly fearful individuals (e.g., an *increase* in COP amplitude [6], rather than the typically observed decreased amplitude [1]). Although there is observational cross-sectional evidence that children with *generalised* anxiety present with greater amplitude COP movements compared to typically developing children [20], these findings cannot infer the direct effect of standing at height on children’s postural control. There is therefore a clear need to explore how standing at height (and the resultant emotional response) directly influences balance control in children. This will enable us to better understand how the high levels of fear and anxiety about falling that occur in children with movement disorders (e.g., developmental coordination disorder [DCD] [21]) affect these individuals’ postural control, aiding the development of effective interventions.

The current study investigated the effects of standing at height on emotional and balance responses in children. To confirm previously reported age-related differences in postural control with height [10], we also compared the responses in children to a cohort of healthy young and older adults. We hypothesised (i) that standing at height would lead to a postural stiffening response (increased frequency and reduced amplitude of COP displacements [1]) in young and older adults, whilst (ii) children would demonstrate an increase in both the frequency and amplitude of COP displacements under the elevated height conditions (i. e., a pattern of potentially maladaptive behaviour that is more typical of a stronger, more robust fear response [6]). Based on previous work [15], we predicted that all groups would exhibit a higher COP sample entropy (indicative of enhanced ‘complexity’ of postural adjustments) when balance safety is threatened, but that this increase would be greatest in the children given that it is strongly associated with the level of fear experienced [8].

## 2 Methods

### 2.1 Participants and sample size estimation

Previous research has reported moderate to large magnitude effect sizes ($\eta^{p2}=0.12$ for an age × condition interaction for MPF) during standing at height conditions compared to baseline (Ground) in young compared to older adults [10]. Power analysis (G*Power, v3.1.9.4) showed that for a repeated measures analysis of variance (ANOVA) analyses a minimum of 42 participants (*n* = 14 per age group) would be required to obtain 80% power (standardised medium effect size, *f*=0.25, *p* = .05) when conducting a 2 (within-subject; Ground vs. Height) × 3 (between-subject; children vs. young vs. older adults) way mixed-model ANOVA. Ninety-eight participants volunteered for this study (Table 1). Children (8 – 11 years, *n* = 38) were recruited from their primary schools in the county of Warwickshire, United Kingdom. Young (18 – 35 years, *n* = 45) and older (65 – 80 years, *n* = 15) adults were recruited from the host institution’s student population and local community groups, respectively. All participants were free from any musculoskeletal dysfunction, neurological impairment, orthopaedic pathology, or special educational needs (e.g., attention deficit hyperactivity disorder [ADHD]). The experimental procedures were carried out in accordance with the standards outlined in the Declaration of Helsinki (1964) and the study received approval by the institutional ethics committee. Prior to conducting the experiment, all participants (as well as the children’s parents) gave their written informed consent.

### 2.2 Protocol

**Fig. 1:**
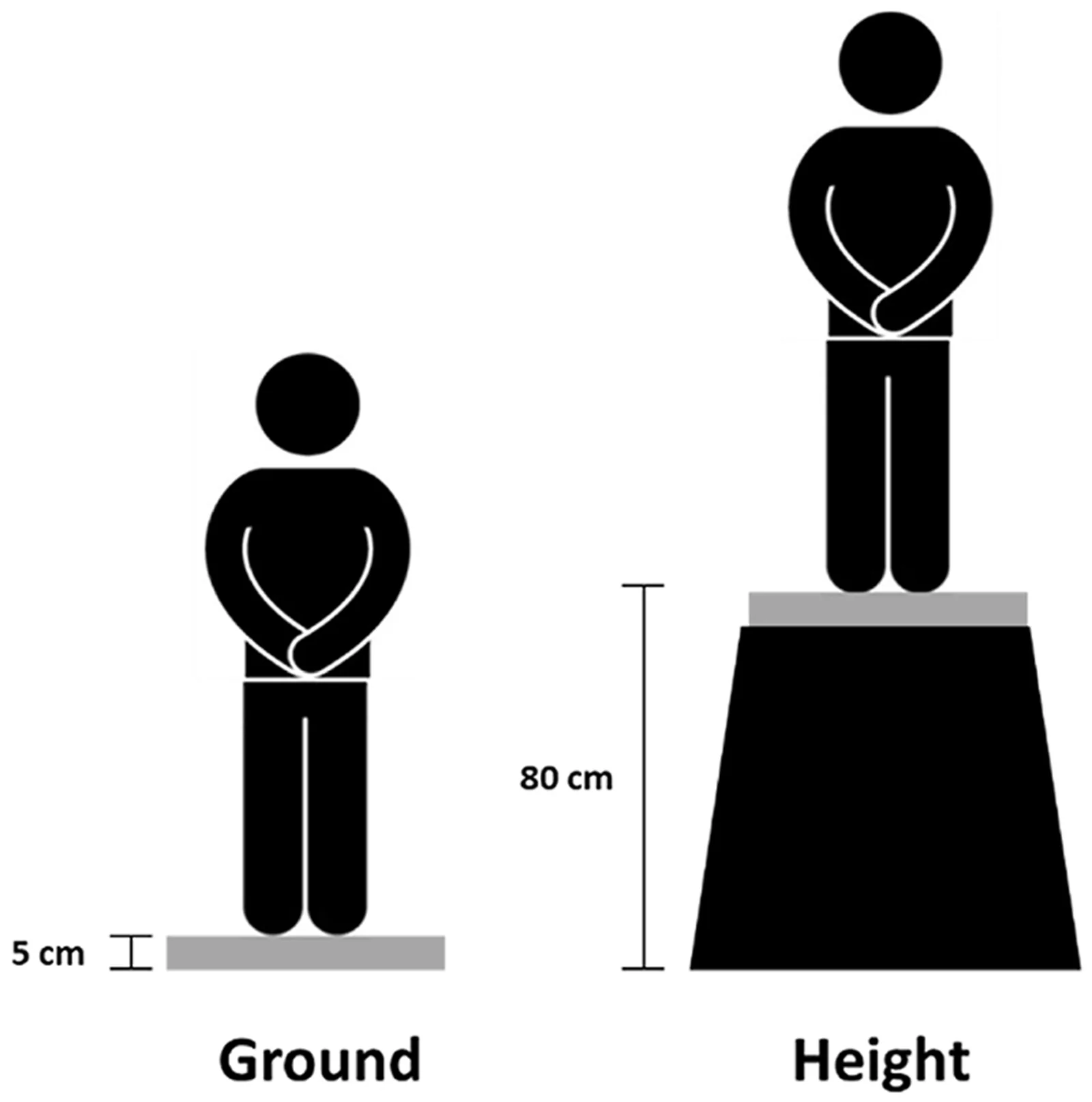
Schematic diagram of the Ground and Height conditions. Note, participants completed all trials without a safety harness.

Participants completed bipedal stance (feet together, narrow stance) balance tasks while standing with their toes positioned at the anterior edge of a force platform (AMTI, AccuGait, Watertown, MA; dimensions 50 × 50 × 5 cm) (Fig. 1). Participants completed a single 60-s trial under the following conditions: on the floor (Ground) and Height (80 cm raised surface). During the Height condition, the 5 cm high force platform was placed on a podium measuring 50 cm long, 50 cm wide and 75 cm high (Fig. 1), resulting in an 80 cm raised surface. Participants always completed the Ground followed by the Height condition. During trials, participants were asked to stand quietly and avoid movement while gazing at a fixation point 3 m away from the force platform. Children were provided with the additional clarification of ‘standing quietly *like a soldier*’ to ensure adequate comprehension of the ‘stand quietly’ task instructions. Participants kept their hands clasped in front of the body at the waist [22]. All trials were completed without a safety harness, and participants were barefoot. To ensure adequate familiarisation, each participant completed a 30-s practice trial at ground level.

### 2.3 Assessment of emotional state outcomes

Immediately prior to each trial (i.e., while standing in position) participants rated how confident they were that they could maintain their balance and avoid a fall using a visual analogue scale (VAS) from 0 (“not at all confident”) to 10 (“completely confident”) [6,23]. Immediately following each trial, participants rated the level of fear of falling they experienced during the trial itself from 0 (adults; “not fearful at all”, children; “not scared”) to 10 (adults; “completely fearful”, children; “really scared”) [7,22]. Participants were then asked to rate their degree of instability during the trial using a 0 – 10 VAS; where, for adults, 0 corresponded to being “completely steady” and 10 “so unsteady that I would fall” [7,9]. For children we instead asked, “how wobbly did you feel”, where 10 corresponded to being “really wobbly” and 0 “really steady”. Note, following pilot testing with 5 children (aged 7 – 9 years) and discussions within the research group, the descriptions of the scoring for these items were simplified for the children to avoid any confusion.

### 2.4 Assessment of postural control outcomes

Ground reaction force data were sampled at 100 Hz (Netforce, AMTI, Watertown, MA) and low-pass filtered (5 Hz) offline with a bidirectional, second-order Butterworth filter. All COP parameters were calculated with respect to the COP mean position [4,7,9,11,12,22,24]. We assessed the amplitude and frequency of postural adjustments by calculating the root mean square (RMS) and mean power frequency (MPF; mean frequency in power spectrum after fast Fourier transformation) of the COP [4,7,9,11,12,22,24]. Given that the platform edge was anterior to participants, all analyses were confined to anterior-posterior (AP) direction, as per previous research [1,8,11]. Higher frequency, coupled with reduced amplitude, of COP displacements is thought to reflect a ‘postural stiffening’ response [1]. We also analysed the complexity (i.e., regularity) of the COP signal by calculating sample entropy (SampEn). For static balance tasks, higher values reflect more ‘complex’ and irregular postural adjustments and is believed to reflect a more automatic (i.e., less consciously processed/controlled) postural control strategy [25,26]. SampEn in the AP direction was calculated on filtered data using a custom MATLAB script (Mathworks, United States) using the following calculations [15,27]:

$$\;SampEn\;=(m,r,N)=-\log\left(\frac{A}{B}\right)$$

where, m is the length of the sequences to be compared, r is the tolerance value for accepting matches, N is the length of the data, and A/B are defined as follows:

$$\begin{array}{c} A=\left\{\frac{\left(n-m-1)(n-m\right)}{2}\right\}A^{m}(r)\\ B=\left\{\frac{\left(n-m-1)(n-m\right)}{2}\right\}B^{m}(r) \end{array}$$

where, *A*^*m*^(*r*) is the probability that sequences match for m + 1 points, and *B*^*m*^(*r*) is the probability that sequences match for m points. We optimised the parameter settings required for the SampEn calculation, resulting in the use of m= 3 and r = 0.25 [28,29].

### 2.5 Statistical analyses

Data were analysed using SPSS version 25.0 (IBM Inc., Chicago, IL). For all analyses, assumptions of normality (Shapiro–Wilk Test) and homogeneity of variance/sphericity (Mauchly Test) were checked and met prior to conducting parametric analyses. A series of mixed-model two-way ANOVAs were undertaken to test for the within-subject effects of standing at height (Ground vs. Height) and age (children vs. young vs. older adults]). Where significant interactions or main effects were detected, post-hoc analyses using Bonferroni-adjusted *α* determined the location of any differences. For ANOVA, effect sizes are reported as partial eta-squared value (η2) and interpreted as negligible (≤0.01) small (0.01 – 0.06), medium (0.06 – 0.14) or large (≥0.14) magnitude effects. Cohen’s *d* is reported for pairwise comparisons and were interpreted as trivial (0–0.19), small (0.20–0.49), moderate (0.50–0.79), and large (>0.80). The alpha value was a priori set at *p* < 0.05 for all tests.

## 3 Results

Table 2 presents the mean ± SD values and Table 3 presents the ANOVA outputs for all assessed variables.

### 3.1 Emotional state outcomes

#### 3.1.1 Balance confidence

**Fig. 2:**
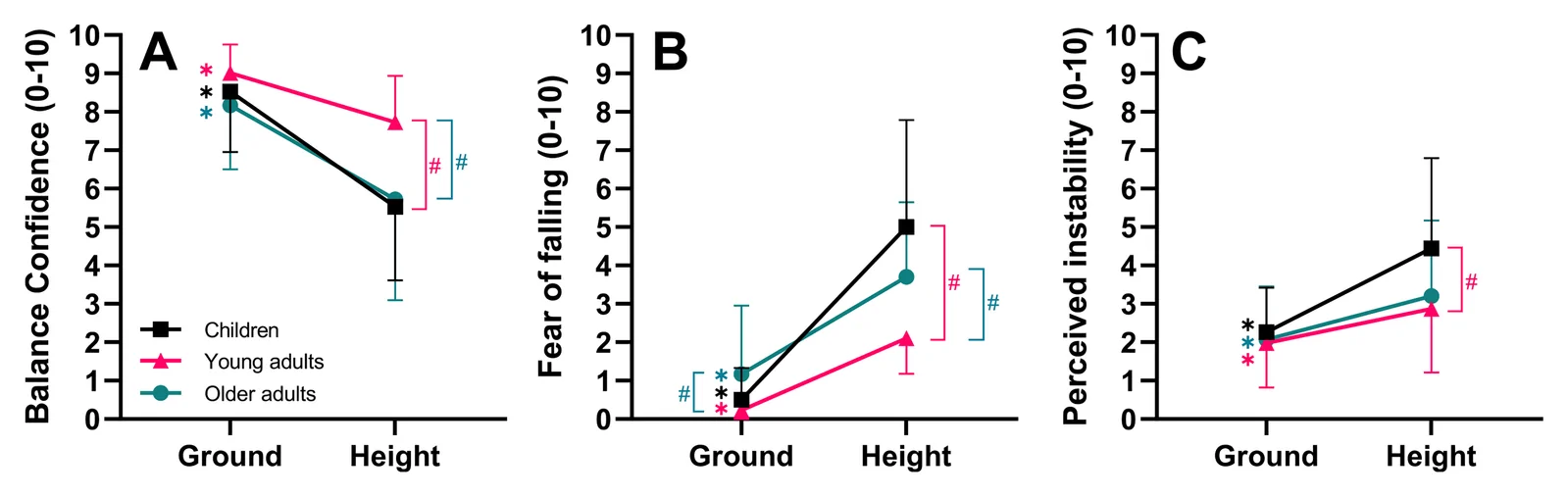
Mean ± SD for self-reported outcomes for children (black), young (pink) and older (blue) adults. An asterisk (*) represents a statistically significant difference to Height (p < .05). A hash (#) represents a significant post-hoc difference between groups (p < .05).

There was a significant main effect of both condition (p < .001) and group (p < .001), as well as a significant interaction between the two (p < .001), with respect to balance confidence. Post-hoc tests revealed a significant decrease in balance confidence from Ground to Height in children (d=1.71), young (d=1.28) and older (d=1.12) adults (all p < .001) (Fig. 2A). Balance confidence during Height was also significantly lower in children (p < .001, d=1.37) and older adults (p < .001, d=2.40), when compared to young adults.

#### 3.1.2 Fear of falling

There was a significant main effect of both condition (p < .001) and group (p < .001), as well as a significant interaction between the two (p < .001), with respect to fear of falling. Post-hoc tests revealed a significant increase in fear of falling from Ground to Height in children (d=2.19), young (d=2.61) and older (d=1.36) adults (all p < .001) (Fig. 2B). Fear of falling during Height was significantly higher in children (p < .001, d=1.39) and older adults (p = .026, d=1.05), compared to young adults. Fear of falling was also greater in older compared to young adults during the Ground condition (p = .002, d=0.72).

#### 3.1.3 Perceived instability

There was a significant main effect of both condition (p < .001) and group (p < .001), as well as a significant interaction between the two (p < .001), with respect to perceived instability. Post-hoc tests revealed a significant increase in perceived instability from Ground to Height in children (p < .001, d=1.18), young (p < .001, d=0.62) and older (p = .008, d=0.66) adults (Fig. 2C). Perceived instability during Height was also significantly higher in children compared to young adults (p = .002, d=0.78).

### 3.2 Postural control outcomes

#### 3.2.1 Centre of pressure amplitude (RMS)

**Fig. 3:**
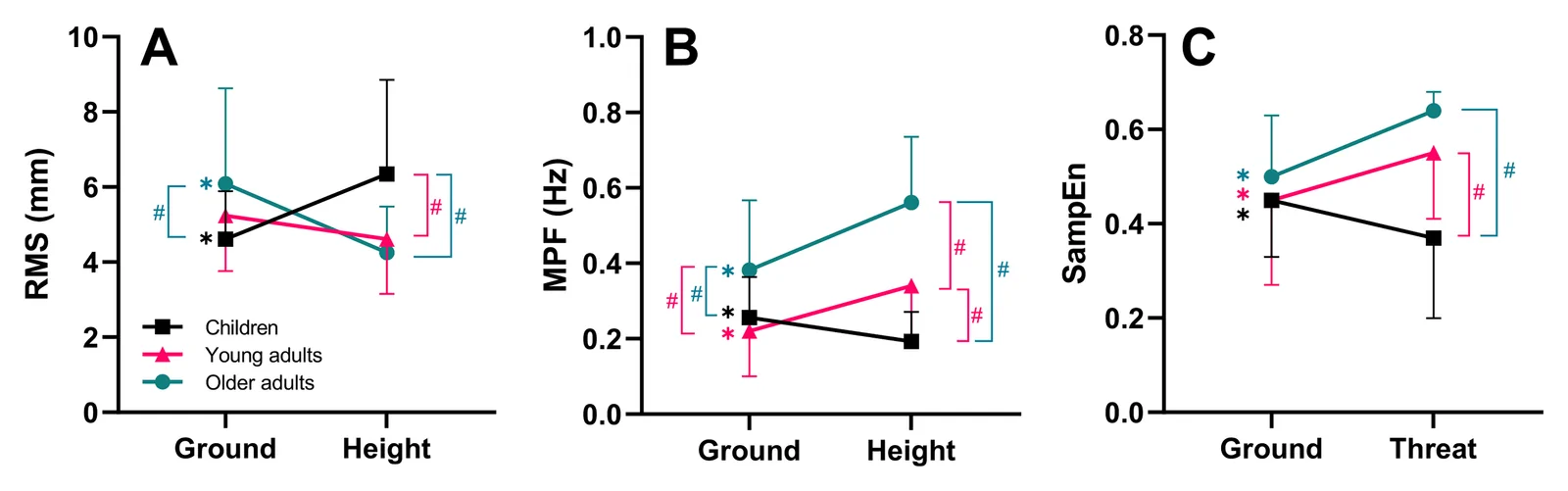
Mean ± SD for postural control outcomes for children (black), young (pink) and older (blue) adults. An asterisk (*) represents a statistically significant difference to Height (p < .05). A hash (#) represents a significant post-hoc difference between groups. RMS; Root mean square, MPF; Mean power frequency, SampEn; Sample entropy.

There was no main effect of group (p = .192) or condition (p = .341), but there was a significant interaction between the two p < .001), with respect to the RMS. Post-hoc tests revealed a significant increase in RMS from Ground to Height in children (p < .001, d=0.87). In contrast, there was a significant *decrease* in RMS during Height for older adults (p = .002, d=0.92) (Fig. 3A). Young adults also tended to exhibit a decrease in RMS, although this did not reach statistical significance when Bonferroni corrections were applied (p = .060, d=0.43). RMS during Height was also significantly higher in children compared to both young (p < .001, d=0.85) and older adults (p = .002, d=1.06).

**Fig. 4:**
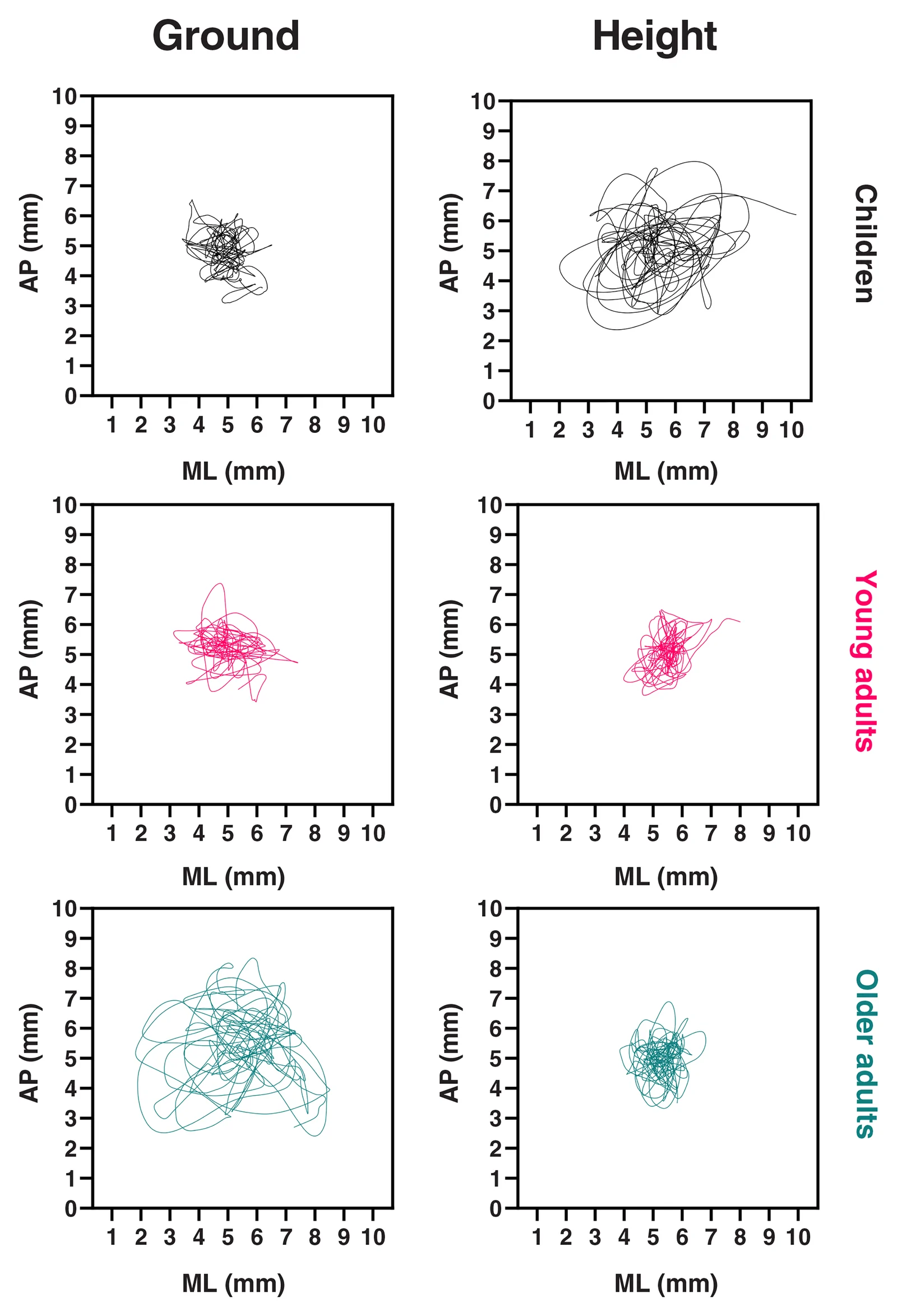
Representative centre of pressure data in one child (black), young (pink) and older (teal) adult during Ground and Height conditions. AP; anterior-posterior, ML; medio-lateral.

RMS was also lower during Ground in children compared to older adults (p = .010, d=0.73). Fig. 4 shows an example illustration of the COP data obtained in one representative participant from each age group, standing under Ground and Height conditions.

#### 3.2.2 Centre of pressure frequency (MPF)

There was a significant main effect of both condition (p < .001) and group (p < .001), as well as a significant interaction between the two (p < .001), with respect to MPF. Post-hoc tests revealed a significant decrease in MPF from Ground to Height in children (p = .017, d=0.67). In contrast, a significant increase in MPF during Height was observed in both young (p < .001, d=0.94) and older adults (p < .001 d=1.00) (Fig. 3B). MPF during Height was significantly lower in children compared to both young (p < .001, d=1.37) and older adults (p = .002, d=2.74). MPF was significantly higher in older compared to young adults during both Ground (p = .001, d=1.02) and Height (p < .001, d=1.41). MPF was similarly greater in older adults compared to children (p = .004, d=0.83) during Ground.

#### 3.2.3 Centre of pressure complexity (SampEn)

There was a significant main effect of both condition (p < .001) and group (p = .008), as well as a significant interaction between the two (p < .001), with respect to SampEn. Post-hoc tests revealed a significant decrease in SampEn from Ground to Height in children (p = .004, d=0.55). In contrast, a significant increase in SampEn from Ground to Height was observed in both young (p < .001, d=0.57) and older adults (p = .002, d=1.44) (Fig. 3B). SampEn during Height was significantly lower in children compared to both young (p < .001, d=1.12) and older adults (p < .001, d=2.21).

## 4 Discussion

This is the first study to compare emotional and behavioural responses to standing at height in children to different-aged adults. Consistent with our hypothesis and previous research [1–12], standing at height led to an increase in both the frequency and complexity, and a decrease in the amplitude, of COP displacements in both young and older adults. These findings are indicative of a postural stiffening strategy, whereby stiffness of the ankle joint is increased to tighten control of the centre of mass within the limits of the base-of-support [1]. In contrast, children showed an increase in amplitude and decrease in both the frequency and complexity of COP, suggestive of a potentially maladaptive postural strategy (given that this strategy would move their centre of mass closer towards the edge of the raised platform). As we observed a lack of significant difference in the strength of the emotional response when comparing children to older adults, the differential behavioural patterns to standing at height in children do not appear to be driven solely by differences in the strength of the psychological response to the height manipulation (as we would not have expected behavioural differences between children and older adults if so). These novel observations instead indicate that incomplete maturation of postural control mechanisms in children may lead to a maladaptive behavioural response when standing in a hazardous situation.

In broad agreement with previous work [4,10,16,17], our observations point towards a similar overall pattern of behavioural responses to height-induced threat in young and older adults. In both groups, the threat manipulation resulted in a significant increase in COP frequency. This occurred in conjunction with a decrease in COP amplitude (although this did not reach statistical significance for the young adults). We interpret these collective findings to imply a protective adaptation from the central nervous system to tighten the control of the COM in situations where safety is threatened by large amplitude postural adjustments [1]. In line with previous work, standing at height also led to significant increases in sample entropy [7,8,15], indicating a more complex and irregular COP signal.

The present study represents the first investigation to explore the influence of standing at height on the control of upright stance in children. In partial support of our hypothesis, children demonstrated a differential postural strategy to adults when exposed to this hazardous condition. That is, children showed an increase in amplitude and a decrease in both the frequency and complexity (SampEn) of COP movements. Similar patterns of increased COP amplitude when standing at height have been observed previously in highly fearful young adults [6]. However, the differential behavioural responses to standing at height in children is unlikely a result of them being less confident/more fearful as there were no differences in emotional state between children and older adults (despite marked differences in behavioural responses between these two groups).

Instead, one possible account for these observations is the incomplete maturation of sensorimotor systems. For instance, research has shown that unlike somatosensory or visual systems, vestibular (reflex) function does not appear to reach adult-like functionality until teenage years [30,31]. Consequently, children experience greater disturbances to postural stability compared to adults during conditions that rely predominantly on vestibular cues for balance (e.g., conditions 5 and 6 of the Sensory Organisation Test [32,33]), suggesting an impaired ability to select and reweight appropriate sensory channels for proper orientation. We therefore propose that children may have been unable to utilise the threat-related vestibular gain (due to incomplete maturation of vestibular function) to “tighten” their postural control in the same way that young and older adults are equipped to do so. Lower COP complexity (SampEn) has been consistently associated with greater conscious attention directed towards balance [25,26,34]. SampEn is known to increase when balance safety is threatened, indicating a more ‘automatic’ mode of postural control [7,8,15]. This is perhaps contradictory, given that height-induced threat is known to also increase conscious attention directed towards balance [9,23,35]. But recent research suggests that during conditions where balance safety is threatened, fearful individuals will nonetheless use conscious strategies to constrain (or, ‘drive down’) threat-related increases in SampEn [7]. This serves to prevent postural control mechanisms from becoming too “random to properly command balance” [28, p. 13]. The significant decreases in SampEn observed for the children when standing at height may therefore reflect an alternative (consciously activated) compensatory strategy. Future work should look to replicate these findings and also assess threat-evoked changes in vestibular processing across age groups to confirm these hypotheses.

We observed a stronger emotional response (i.e., greater fear of falling and lower balance confidence) to the raised platform in both children and older adults, compared to the young adults. In older adults, the greater fear of falling likely reflects their generalised concerns about falling and the recognition of their risk for experiencing an injury if they were to fall, leading to a stronger fear response [36,37]. In contrast, the stronger fear of falling response in children more likely reflects the still developing emotional regulation circuits [38]. As such, when compared to young adults, children may have been less able to inhibit responding emotionally to the threat, resulting in a stronger and more robust fear response. It is important to emphasise that the verbal descriptions of the scoring for the fear question were simplified for children following pilot testing. Therefore, we cannot to exclude the possibility that differential fear scores observed in children and adults were a consequence of different interpretations to the question, rather than age-related differences in the emotional response itself. However, since all emotional outcomes followed similar patterns in all groups, we deem that such simplification likely exerted minimal effect on the data presented. Nonetheless, future studies should look to clarify this point.

An open question concerns whether a similar pattern of results extend to other types of threat manipulations. Although increased MPF and reduced RMS of COP has consistently been reported when exposed to height induced threats [1], when standing in anticipation of receiving an unpredictable perturbation, participants instead demonstrate increases in *both* amplitude and frequency of COP displacements [16]. Therefore, the observations here should only be generalised to the specific height-induced threat manipulation employed in this study. Future studies should therefore examine whether the age-related differences reported here can be generalised to other types of threat stimuli (e.g., anticipation of receiving an unpredictable surface translation).

## 5 Conclusion

This study demonstrates distinct threat-induced postural control strategies in children compared to (young and older) adults. Young and older adults exhibited a (potentially protective) “stiffening” response to a height-induced threat, characterised by increased frequency but reduced amplitude COP movement. In contrast, children demonstrated the opposite behaviours, exhibiting *reduced* frequency and *increased* amplitude COP movement, reflecting instead a potentially maladaptive postural adaptation strategy. These observations expand upon existing postural threat research in adults, providing important new insight into understanding how children respond to standing in a hazardous situation. Such findings have implications for understanding and intervening to target the high levels of fear and anxiety about falling that occur in children with movement disorders e.g. developmental coordination disorder (DCD [21]).

## Figures and Tables

**Table 1:** Participant characteristics.

	Children	Young adults	Older adults
Sample size (*n*)	38	45	15
Sex (females; *n*)	17	19	7
Age (years)	9.7 ± 0.8	21.8 ± 4.0	73.3 ± 5.0
Body height (m)	1.42 ± 0.08	1.74 ± 0.09	1.67 ± 0.14
Body mass (kg)	38.8 ± 12.3	72.5 ± 14.6	70.3 ± 16.5
Body mass index (BMI)	19.1 ± 5.2	23.8 ± 3.8	25.2 ± 4.4

**Table 2:** Mean ± SD for all emotional state and postural control outcomes for children, young and older adults during Ground and Height conditions.

	Children			Young adults			Older adults	
	Ground	Height		Ground	Height		Ground	Height
Confidence (0-10)	8.5 ± 1.6	5.5 ± 1.9		9.0 ± 0.7	7.7 ± 1.2		8.2 ± 1.7	5.7 ± 2.6
Perceived instability (0-10)	2.3 ± 1.2	4.5 ± 2.3		2.0 ±1.2	2.9 ± 1.7		2.1 ± 1.4	3.2 ± 2.0
Fear of falling (0-10)	0.5 ± 0.8	5.0 ± 2.8		0.2 ± 0.4	2.1 ± 0.9		1.2 ± 1.8	3.7 ± 1.9
RMS (mm)	4.61 ± 1.28	6.35 ± 2.51		5.24 ± 1.48	4.61 ± 1.46		6.09 ± 2.54	4.26 ± 1.22
MPF (Hz)	0.26 ± 0.11	0.19 ± 0.08		0.22 ± 0.12	0.34 ± 0.13		0.38 ± 0.19	0.56 ± 0.17
SampEn (A.u)	0.45 ± 0.12	0.37 ± 0.12		0.45 ± 0.18	0.55 ± 0.14		0.50 ± 0.13	0.64 ± 0.04

RMS; Root mean square, MPF; Mean power frequency, SampEn; Sample entropy

**Table 3:** Main and interaction effects of the repeated measures ANOVA for emotional state and postural control outcomes.

	*F*	*P*	$\eta_{\text{p}}^{2}$
**Balance confidence**			
Group *(children* vs. *young* vs. *older)*	13.111	**.001**	.216
Condition *(Ground vs. Height)*	154.657	**.001**	.619
Group × Condition interaction	12.265	**.001**	.205
**Fear of falling**			
Group (*children* vs. *young* vs. *older*)	19.394	**.001**	.290
Condition (*Ground* vs. *Height*)	176.790	**.001**	.650
Group × Condition interaction	18.651	**.001**	.282
**Perceived instability**			
Group (*children* vs. *young* vs. *older*)	4.498	**.001**	.087
Condition (*Ground* vs. *Height*)	59.339	**.001**	.384
Group × Condition interaction	6.976	**.001**	.128
**RMS**			
Group (*children* vs. *young* vs. *older*)	1.680	.192	.034
Condition (*Ground* vs. *Height*)	.917	.341	.010
Group × Condition interaction	18.258	**.001**	.278
**MPF**			
Group (*children* vs. *young* vs. *older*)	37.511	**.001**	.441
Condition (*Ground* vs. *Height*)	18.003	**.001**	.159
Group × Condition interaction	18.053	**.001**	.275
**SampEn**			
Group (*children* vs. *young* vs. *older*)	10.622	**.001**	.183
Condition (*Ground* vs. *Height*)	7.225	**.008**	.071
Group × Condition interaction	14.551	**.001**	.234

ηp2 are interpreted as negligible (≤.0.01) small (0.01 – 0.06), medium (0.06 – 0.14) or large (≥0.14) magnitude effects. Bold values indicate statistically significant effects.
